# Phase-Specific Vocalizations of Male Mice at the Initial Encounter during the Courtship Sequence

**DOI:** 10.1371/journal.pone.0147102

**Published:** 2016-02-03

**Authors:** Yui K. Matsumoto, Kazuo Okanoya

**Affiliations:** Department of Life Sciences, Graduate School of Arts and Sciences, The University of Tokyo, 3-8-1 Komaba, Meguro-ku, Tokyo,153–8902, Japan; Claremont Colleges, UNITED STATES

## Abstract

Mice produce ultrasonic vocalizations featuring a variety of syllables. Vocalizations are observed during social interactions. In particular, males produce numerous syllables during courtship. Previous studies have shown that vocalizations change according to sexual behavior, suggesting that males vary their vocalizations depending on the phase of the courtship sequence. To examine this process, we recorded large sets of mouse vocalizations during male–female interactions and acoustically categorized these sounds into 12 vocal types. We found that males emitted predominantly short syllables during the first minute of interaction, more long syllables in the later phases, and mainly harmonic sounds during mounting. These context- and time-dependent changes in vocalization indicate that vocal communication during courtship in mice consists of at least three stages and imply that each vocalization type has a specific role in a phase of the courtship sequence. Our findings suggest that recording for a sufficiently long time and taking the phase of courtship into consideration could provide more insights into the role of vocalization in mouse courtship behavior in future study.

## Introduction

Many animals use vocalizations for communication, and vocalizations can have a variety of acoustic characteristics depending on the context or behavior. Ultrasonic vocalizations often occur during interactions between individual mice. Several studies have investigated ultrasonic vocalizations during male–female interactions, wherein they focused on the number and types of syllables involved [1–3]. During male–female interactions most vocalizations are emitted by the male mouse [4–6], and females are attracted to male vocalizations [7–11]. Therefore, it seems likely that these ultrasonic vocalizations of male mice play a role in courtship.

Courtship vocalizations consist of various syllable types [2, 12] that can be divided into approximately 10 major categories based on duration, frequency modulation and the presence or absence of frequency jumps [13–16]. The proportion of syllable types used changes during the development of an individual [12, 17]. Adult mice typically express similar patterns of syllable use, although these patterns can vary between strains [9, 11, 14]. However, the patterns of vocalizations produced by adults do not necessarily remain constant throughout a social interaction, but can change depending on behavior, especially mounting behavior. White et al. [4] demonstrated that male mice emit 40 and 70 kHz vocalizations during their interactions with females, and the vocalizations change with the onset of mounting and intromission behavior. Similarly, harmonic calls have been associated with mounting behavior in previous studies [18, 19]. In addition, a study comparing male courtship vocalizations between amygdala-lesioned and sham mice found that lesioned mice exhibited a lower incidence of mounting behavior and longer syllables, especially harmonic syllables, than sham mice [20]. Syllables containing harmonics are therefore closely associated with mounting behavior. In addition, syllable duration may vary throughout an interaction. Following amygdala lesioning, the proportion of short syllables increased with increasing sniffing behavior [20], suggesting that short syllables are produced during sniffing behavior and supporting the supposition that mice emit different vocalizations depending on context. Furthermore, mice continuously produce short syllables in the early phase of interaction [20]. However, no study has quantitatively examined mouse usage of vocalizations over the course of a courtship interaction. Courtship behavior consists of several phases, from the encounter with the female through ejaculation. It is likely that males produce several patterns of vocalizations depending on the courtship behavior or the phase of interaction; however, the relationship between the changes in mouse vocalizations and the phases of the courtship sequence is not clear. The lack of understand stems from the ambiguity in the turning point in vocal communication that precipitates shifts to the next stage for mice. In this study, we assessed differences in the acoustic features and proportions of ultrasonic vocalization patterns of male mice during the courtship sequence. To accomplish this, once mice exhibited sniffing or mounting behavior we recorded the male’s vocalizations for 10 min and compared vocalizations over time and among the early, middle and late phases of interaction.

## Methods

### Animals

All experimental procedures were approved by the Animal Experiment Committee of the University of Tokyo. Experimental animals were C57BL/6Ncr mice aged 10–19 weeks (Japan SLC, Hamamatsu, Japan). Males (n = 16) were housed individually throughout the experiment in Plexiglas cages (16 cm × 23 cm × 12 cm), and females (n = 27) were housed in groups of four or five. Cages were kept in a controlled environment at 22 ± 2°C, with a 12 h light/dark cycle (lights off at 1300 h). Food and water were provided *ad libitum*. After the study, animals were used for other experiments.

In order to control the estrus cycle, females were ovariectomized and two Silascon tubes (Inside diameter, 1.0 mm; Outside diameter, 2.0 mm × 10.0 mm; Kaneka Medix, Osaka, Japan) containing β-estradiol 3-benzoate (approximately 98%, 2 molL^-1^; Wako Pure Chemical Industries, Osaka, Japan) were subcutaneously implanted at the nape the neck a week before introduction to males. Implantation was performed under anesthesia using sodium pentobarbital (64.8 mgmL^-1^, quintuple dilution).

### Recording of ultrasonic vocalizations and courtship behavior

All recordings were made in a sound-attenuating chamber (MC-050, Muromachi Kikai, Tokyo, Japan). To decrease noise from animal movement, a silicon rubber cover was placed on the bottom of the test cage. Males were acclimatized to the recording cage (15 × 15 × 15 cm) during 1400−2000 h for five days prior to the recording test. During recording, a male mouse was placed in this cage, and a female was added 10 min later. Interactions were monitored by means of audio and video recording for 10 min, beginning when the female was placed in the cage. These experiments were conducted on seven consecutive days, once per day, during the dark cycle (1400−2000 h). The same male–female pair was never placed together more than once across all tests, and the order of mice was random for the dairy recording. The microphone (UltraSoundGate CM16/CMPA; Avisoft Bioacoustics, Berlin, Germany) was sensitive to sounds in the range of 10–180 kHz. Vocalizations were recorded using Avisoft RECORDER software with a sampling rate of 300 kHz. The camera (Adafruit TTL serial camera; Adafruit industries, New York, NY, USA) was attached to the top of the sound chamber. The cage was illuminated with a red light. We enumerated two behaviors: sniffing (male’s nose directly in contact with female) and mounting (male places his forelegs on female’s back).

According to previous studies, most vocalizations are produced by the male [4–6], and many of them only occur upon direct contact with a female or fresh female urine [2, 3, 18, 21, 22]. In the present study, males performed most direct body contact (sniffing or mounting) during male–female interactions, and most of the vocalizations occurred at the same time. We therefore assumed that most of the vocalizations we recorded were produced by the males.

### Ultrasound analysis

The vocalization data from the day on which each mouse emitted the greatest number of syllables were used in our analysis. We excluded data from the first day in all cases, because vocalizations were affected by the initial interaction with females and varied considerably among males. Sound spectrograms were generated with a fast Fourier transform length of 256 points and a time-window overlap of 75% (100% frame, Hamming window). The spectrogram was produced at a frequency resolution of 977 Hz and a time resolution of 2 ms. Sounds with frequencies below 35 kHz were removed to reduce the effects of background noise occurring outside the relevant frequency band. Acoustic features (mean syllable duration, root mean square amplitude (RMS), peak frequency, fundamental frequency, bandwidth, and entropy) were measured using “automatic measurements,” a function of SASLab Pro (Avisoft Bioacoustics), after removing sounds of irrelevant activities such as scratching and locomotion.

We classified the vocalizations into 12 types, based on syllable duration and frequency modulation, adapted from previously published criteria [13–16, 23]. Most syllables could be grouped into short (≦ 60 ms) or long (> 60 ms) syllable types, but “One jump” and “Multiple jumps” which contained frequency jumps are widely distributed in duration from short to long. We therefore separated these syllables into two subtypes (short and long) each according to their duration. In previous studies, “short” (shorter than 5 ms) has been considered a syllable type; however, we grouped syllables into short (Upward, Downward, Flat, Chevron, U shape, Wave, One jump short, and Multiple jumps short) and long types (Complex, One jump long, Multiple jumps long, and Harmonics). Syllables classified as “Flat” in this study included most of the “short” syllables. In addition, we classified syllables as “Harmonics” when a harmonic was present. We investigated several acoustic characteristics of each syllable type.

The syllable classification we used is as follows:

**Upward**: syllables with upsweep frequency change (> 5 kHz)
**Downward**: syllables with downsweep frequency change (> 5 kHz)
**Flat**: syllables with minimal frequency change (≤ 5 kHz)
**Chevron**: syllables with upsweep (> 5 kHz) followed by downsweep (> half of the frequency change of the upsweep)
**U shape**: syllables with downsweep (> 5 kHz) followed by upsweep (> half of the frequency change of the downsweep)
**Wave**: syllables two phases and a change in frequency (> 5 kHz)
**Complex**: syllables with three or more phases and changes in frequency (> 5 kHz)
**One jump (short)**: syllables with one frequency jump (lasting < 60 ms)
**One jump (long)**: syllables with one frequency jump (lasting ≥ 60 ms)
**Multiple jumps (short)**: syllables with two or more frequency jumps (lasting < 60 ms)
**Multiple jumps (long)**: syllables with two or more frequency jumps (lasting ≥ 60 ms)
**Harmonics**: syllables with one or more harmonic sounds

**Fig 1:**
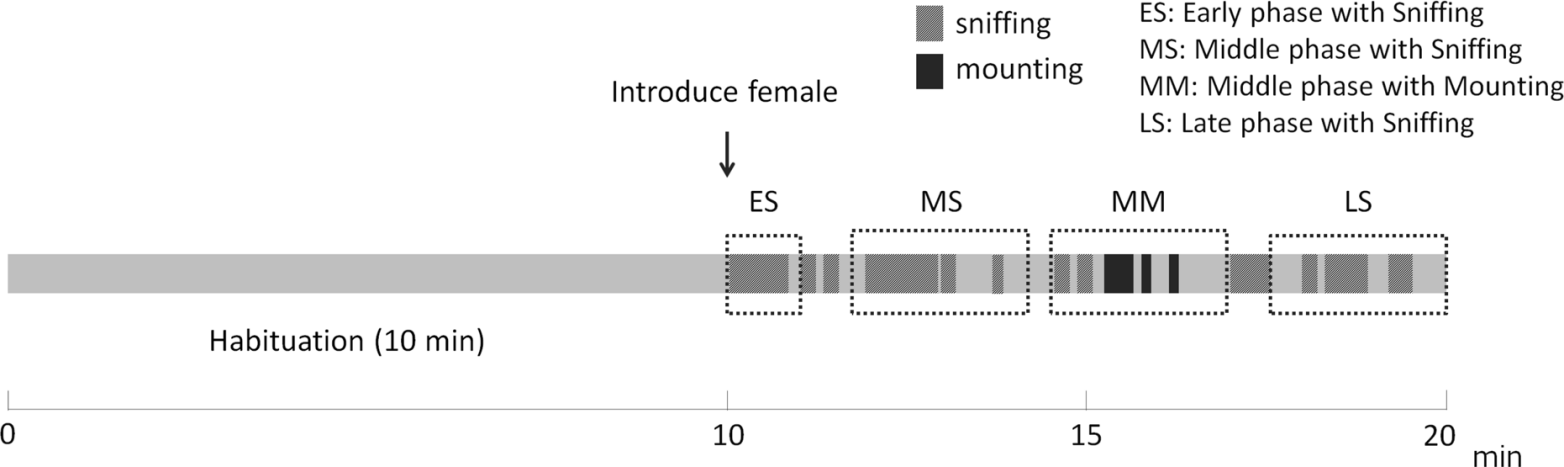
Example of the experimental procedure to record vocalization and behavior. Ten minutes after a male had been placed in a recording cage for habituation, a female was introduced into the cage and ultrasonic vocalizations were recorded for 10 min. We divided the vocalizations into four categories based on behavior and contact time (see text for details).

We investigated the temporal changes in several acoustic features and the number and proportion of each syllable type in relation to behavior. We divided mouse vocalizations into four groups according to contact time and behavior in the courtship sequence (Fig 1): (i) sniffing during the early phase (ES; vocalizations with sniffing during the first minute following introduction of the female); (ii) sniffing during the middle phase (MS; vocalizations with sniffing for 3 min between 1 and 7 min after the introduction of the female); (iii) mounting during the middle phase (MM; vocalizations with sniffing and three or more instances of mounting behavior for 3 min between 1 and 7 min after the introduction of the female); and (iv) sniffing during the late phase (LS; vocalizations with sniffing during the last 3 min to the end of recording). Trials in which mice emitted fewer than 100 syllables or showed only 1–2 instances of mounting behavior were excluded from statistical analysis.

### Statistical analysis

Statistical comparisons were conducted by performing one- and two-way analysis of variance (ANOVA) followed by Tukey’s test, Bonferroni’s test, and tests for linear trends using GraphPad Prism software (GraphPad, San Diego, CA, US). For each syllable type, we averaged the mean values for syllable duration, RMS, peak frequency, fundamental frequency, bandwidth, and entropy (n = 16). We compared data on the acoustic features listed above, as well as the total syllable duration per minute, number of syllables per minute, and proportion of each syllable type among the four phases of the courtship sequence. We also examined how several acoustic features (mean syllable duration, number of syllables, total duration) and the proportion of each syllable type changed with contact time. A Kolmogorov-Smirnov test was used to compare the distribution and cumulative percentage frequency of mean syllable duration. We analyzed correlations between long syllables and mounting behavior and between the proportions of several syllable types using a Spearman’s rank correlation test. Multidimensional scaling (MDS) analysis was used to estimate the similarity in the use of vocalizations between syllable types. Each cell contained the average proportion of each syllable type in ES, MS, MM and LS, and Euclidian distances between each syllable type were calculated using R version 3.0.2. These data were displayed on a hierarchical clustering dendrogram and a MDS plot. All data are presented as mean ± standard error of the mean (SEM).

## Results

### Acoustic features of the 12 syllable types

We analyzed 10,665 syllables (666.6 ± 56.46 syllables per animal) classified into the 12 types section based on their acoustic characteristics (Table 1). Mean syllable duration of most categories was short (≦ 60 ms), but Complex, One jump (long), Multiple jumps (long) and Harmonics syllables were longer (> 60 ms), and in many cases Harmonic syllables were particularly long (F (11, 165) = 159.8, *p* < 0.0001). The RMS of these four types was also larger than that of the others (F (11, 165) = 68.48, *p* < 0.0001), especially for the Harmonics syllables (-24.91 ± 0.3302 dB). Bandwidth of the same four categories was smaller (F (11, 165) = 36.93, *p* < 0.0001). Peak (F (11, 165) = 16.65, *p* < 0.0001) and fundamental frequency (F (11, 165) = 36.68, *p* < 0.0001) was lowest in the Harmonics. Syllables could therefore be divided into two major categories: (1) short syllables that had lower RMS and higher peak frequency and bandwidth; and (2) long syllables that had higher RMS and lower bandwidth. Harmonics were long and had the largest RMS and lowest peak and fundamental frequency of all syllable types.

**Table 1:** Acoustic features of the 12 syllable types.

Syllable types / Acoustic features	Duration (ms)	RMS (dB)	Peak frequency (kHz)	Fundamental frequency (kHz)	Bandwidth (Hz)	Entropy
Upward	16.78 ± 1.084	-34.67 ± 0.6413	77.002 ± 1.307	69.296 ± 1.314	2034 ± 34.14	0.1985 ± 0.006237
Downward	24.98 ± 1.509	-35.67 ± 0.7524	75.08 ± 1.717	67.485 ± 1.487	1888 ± 37	0.1982 ± 0.006995
Flat	15.03 ± 1.567	-38.35 ± 1.077	72.75 ± 1.715	68.172 ± 1.537	1819 ± 63.76	0.1898 ± 0.01036
Chevron	41.8 ± 3.116	-31.22 ± 0.8801	77.889 ± 1.705	69.138 ± 1.501	1761 ± 36.31	0.1889 ± 0.005139
U shape	31.92 ± 1.747	-32.69 ± 0.6737	76.808 ± 1.36	69.779 ± 1.48	1744 ± 38.49	0.1864 ± 0.006127
Wave	52.17 ± 2.321	-31.23 ± 0.5268	77.166 ± 1.339	68.538 ± 1.488	1650 ± 3.589	0.1844 ± 0.005296
Complex	101.9 ± 5.563	-28.75 ± 0.6772	74.58 ± 1.145	65.863 ± 1.317	1519 ± 33.21	0.1825 ± 0.00561
One jump short	28.34 ± 1.152	-32.65 ± 0.9466	77.51 ± 1.092	66.305 ± 1.505	1860 ± 31.28	0.1999 ± 0.005457
One jump long	94.81 ± 4.262	-27.68 ± 0.6792	74.283 ± 1.075	64.163 ± 1.018	1519 ± 27.41	0.1814 ± 0.004052
More jumps short	44.04 ± 1.511	-32.7 ± 0.8667	78.643 ± 1.283	64.492 ± 1.562	1733 ± 28.93	0.2089 ± 0.005008
More jumps long	112 ± 5.58	-28.22 ± 0.558	73.884 ± 1.136	60.199 ± 1.328	1530 ± 30.11	0.1992 ± 0.004062
Harmonics	145.7 ± 8.419	-24.91 ± 0.3302	66.728 ± 1.265	49.06 ± 1.239	1485 ± 40.22	0.2102 ± 0.003826

Values shown are the mean ± SEM.

### Changes in mean syllable duration, number of syllables, and total duration

**Fig 2:**
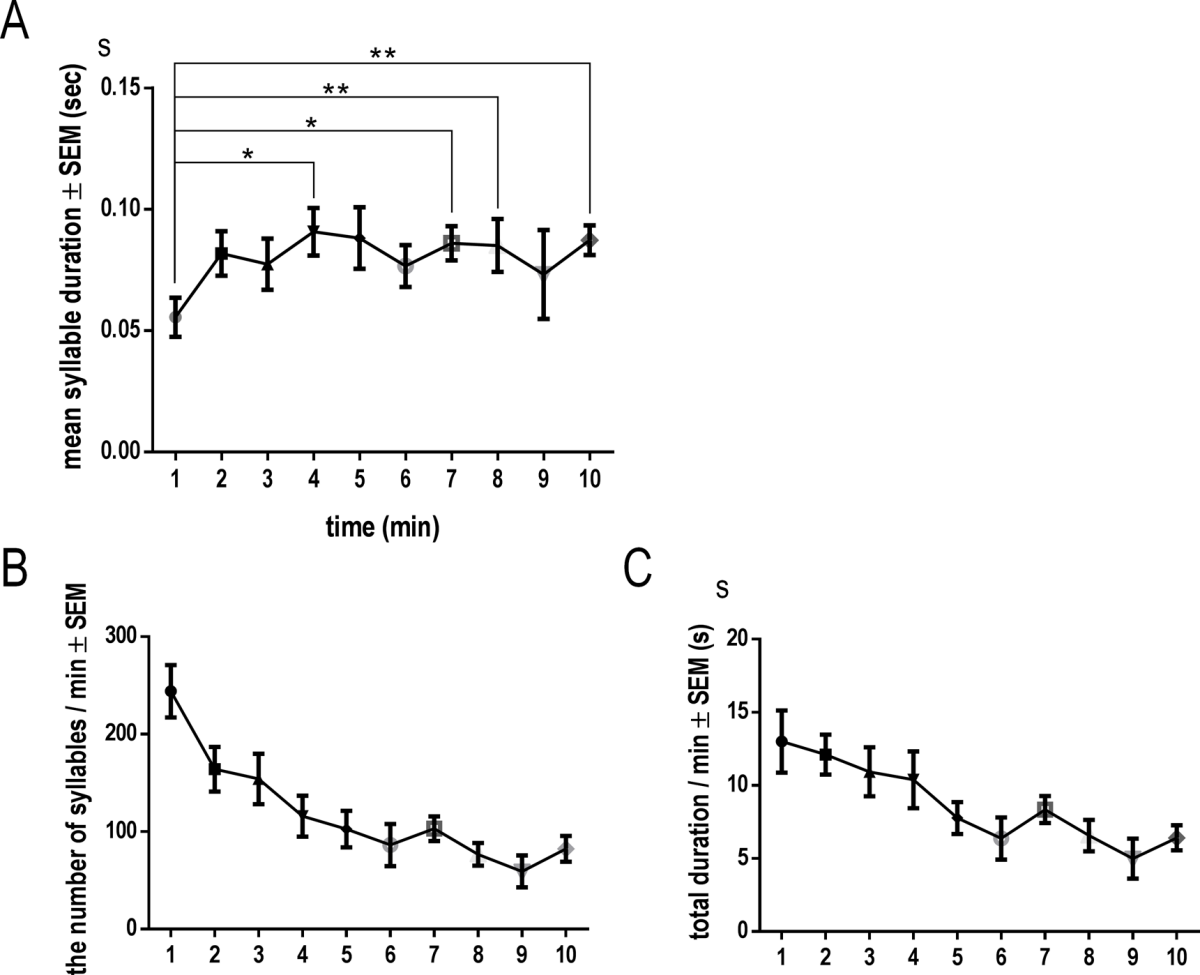
Temporal changes in mouse vocalizations during male–female interactions. Temporal changes in mean syllable duration (A), number of syllables (B) and total duration (C) during male–female interactions for 10 min following introduction of a female. Males showed a greater number of shorter syllables in the first minute after introduction of a female. Data shown are mean ± SEM. Statistical significance is denoted by asterisks: *p < 0.05, **p < 0.01, ***p < 0.001, and ****p < 0.0001.

To examine the relationship between contact time and vocalizations, we investigated temporal changes in several acoustic features (mean syllable duration, number of syllables and total duration) in the vocalizations of 10 males. Mice that did not produce sufficient vocalizations (i.e., those produced less than 10 syllables per min) were excluded from this analysis. Mean syllable duration was shorter during the first 60 s (52.2 ± 7.94 ms) than at four (90.0 ± 8.77 ms), seven (83.9 ± 6.67 ms), eight (83.2 ± 9.96 ms) and 10 min (83.4 ± 6.72 ms) after the introduction of females (F (9, 81) = 10.6, *p* < 0.05; Fig 2A). The number and total duration of syllables decreased with interaction time (Test for linear trends; number of syllables: slope = -7.97, *p* < 0.0001; total duration of syllables: slope = -0.420, *p* < 0.0001; Fig 2B and 2C).

### Differences in acoustic features and proportion of syllable types among courtship phases

We categorized mouse vocalizations according to contact time and behavior. Most animals only performed the sniffing behavior in the early and late phases; we therefore examined the differences among vocalizations emitted during the four phases of the courtship sequence (ES, MS, MM and LS; Table 2). As mentioned above, the number of syllables during the first minute after the introduction of females was the highest in 10 min recording and decreased with interaction time (Fig 2B). Therefore, we analyzed syllables for 1 min period in ES and for 3 min during the other phases (2166–3177 syllables in total). Total duration was longest in MM (14.12± 1.857 s; F (3, 32) = 11.0, *p* < 0.05), followed by that in ES (9.098 ± 1.725 s). Mean syllable duration was greater in MM (86.97 ± 8.438 ms) than in ES (37.75 ± 3.727 ms; F (3, 32) = 8.55, *p* < 0.01). In contrast, the number of syllables was greatest in ES (265.8 ± 25.76; F (3, 32) = 23.2, *p* < 0.05), followed by MM (176.5 ± 30.94). Thus, males produced the greatest number of short syllables in ES, but total duration of vocalizations was longest in MM, as the longest syllables were generated during that phase. Additionally, the spectrographic analysis revealed that vocalizations emitted during ES had the highest fundamental frequency (68.67 ± 1.631 kHz) and those in MM (59.32 ± 2.125 kHz) had the lowest frequency (F (3, 32) = 3.34, *p* < 0.05). Bandwidth (1610 ± 23.08 kHz; F (3, 32) = 8.29, *p* < 0.01) and entropy (0.1867 ± 0.007772; F (3, 32) = 16.6, *p* < 0.05) were also lowest in MM. Thus, males produced sharp sounds with a low fundamental frequency and bandwidth during phases featuring mounting behavior. There were no discernible differences in the eight acoustic parameters between MS and LS, indicating that syllable types during sniffing after the early phase did not change as a function of contact time. In this study, three mice displayed intromission behavior. The frequency of vocalizing, mean syllable duration, and mean break duration (the duration between syllables) were lower during intromission than mounting in all animals (S1 Fig). These results suggest that vocalizations during intromission are different from those during mounting.

**Table 2:** Acoustic features of vocalizations emitted during the four phases of the courtship sequence.

Groups / Acoustic features	Total number of syllables (syllables)	Total duration / min (s)	Number of syllables / min	Mean syllable duration (ms)	RMS amplitude (dB)	Peak frequency (kHz)	Fundamental frequency (kHz)	Bandwidth (Hz)	Entropy
ES	2658 (n = 11)	9.098 ± 1.725	265.8 ± 25.76	37.75 ± 3.727	-32.66 ± 1.213	76.2 ± 1.771	68.67 ± 1.631	1898 ± 34.40	0.2108 ± 0.002995
MS	2664 (n = 10)	4.56 ± 0.5704	88.8 ± 12.11	70.79 ± 7.635	-31.97 ± 0.801	74.898 ± 1.503	64.68 ± 1.932	1813 ± 45.34	0.2214 ± 0.003589
LS	2166 (n = 10)	4.59 ± 0.7116	72.2 ± 8.738	72.72 ± 7.753	-32.16 ± 0.76	75.134 ± 1.669	64.37 ± 1.971	1815 ± 33.41	0.2258 ± 0.002108
MM	3177 (n = 6)	14.12 ± 1.857	176.5 ± 30.94	86.97 ± 8.438	-29.99 ± 1.088	75.669 ± 2.724	59.32 ± 2.125	1610 ± 23.08	0.1867 ± 0.007772

Average scores were compared using a one-way ANOVA followed by Tukey’s test. Data shown are mean ± SEM.

**Fig 3:**
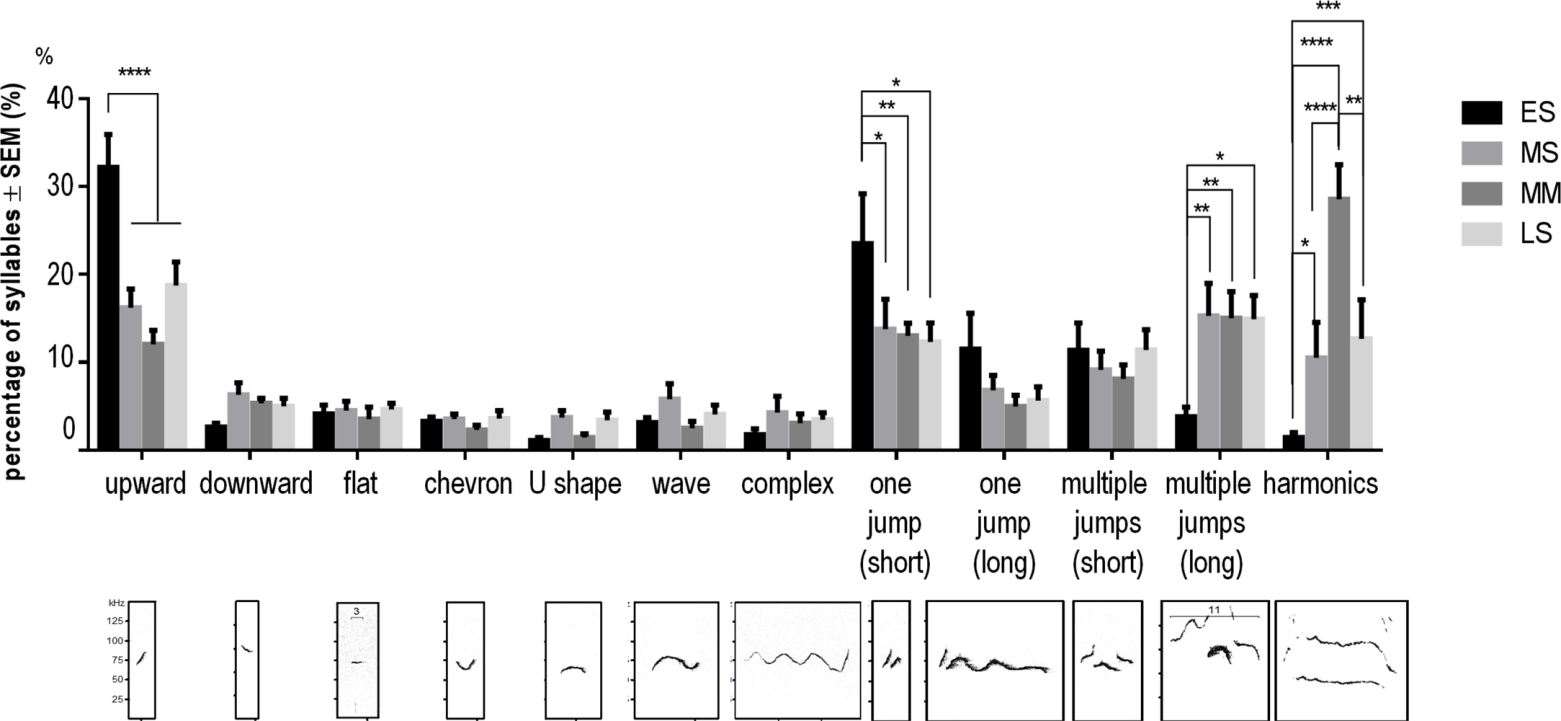
The proportions of each syllable type in the four phases of courtship sequence. The proportions of Upward, One jump (short), Multiple jumps (long) and Harmonics syllables changed noticeably depending on the phase of the courtship sequence. Data shown are mean ± SEM. Statistical significance is denoted by asterisks: *p < 0.05, **p < 0.01 and ****p < 0.0001.

**Fig 4:**
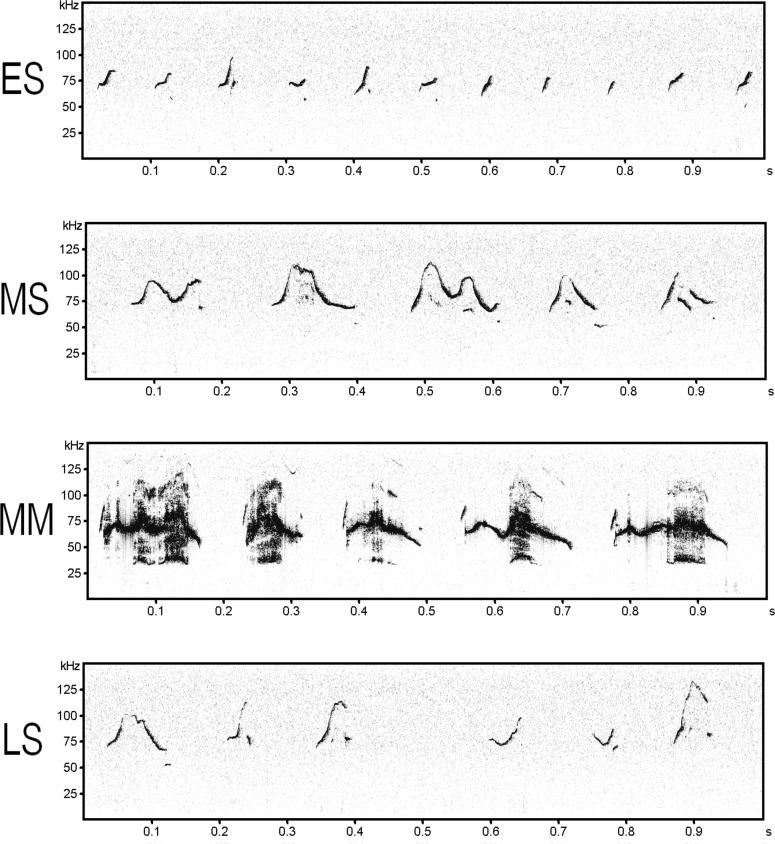
Spectrogram of ultrasonic vocalizations during the ES (A), MS (B), MM (C), and LS (D).

Mice emitted all types of syllables during each phase of courtship behavior, and produced high proportions of Upward (20.9 ± 1.85%), One jump (short) (16.0 ± 2.04%), Multiple jumps (long) (11.9 ± 1.56%) and Harmonics (11.4 ± 2.24%) syllables throughout the interactions (F (11, 456) = 20.6, *p* < 0.0001; Fig 3A). The proportions of these four syllable types differed noticeably among ES, MS (LS), and MM, but this was not true for the other syllables (S1, S2 and S3 Movies). There was a greater proportion of Upward (32.2 ± 3.71%) and One jump (short) (23.5 ± 5.67%) syllables during ES than in other phases (F (3, 35) = 0.238, *p* < 0.05; Figs 3B and 4). In contrast, the proportions of Multiple jumps (long) (3.83 ± 1.09%) and Harmonics (1.44 ± 0.578%) were lower during ES than during other phases (F (3, 35) = 0.238, *p* < 0.05; Fig 3B). In particular, the proportion of Harmonics syllables, defined by long duration and low fundamental frequency, was greatest during MM (28.6 ± 3.94%; Figs 3B and 4). In addition, long syllables (> 0.1 s) were strongly correlated with mounting behavior (*R* = 0.87, n = 15). Thus, males displayed sniffing behavior and produced a considerable number of short syllables for approximately one minute after a female was introduced. On the other hand, males produced long syllables after their initial interaction with females, and a higher proportion of Harmonics throughout mounting behavior.

### Temporal changes in the proportion of syllable types

**Fig 5:**
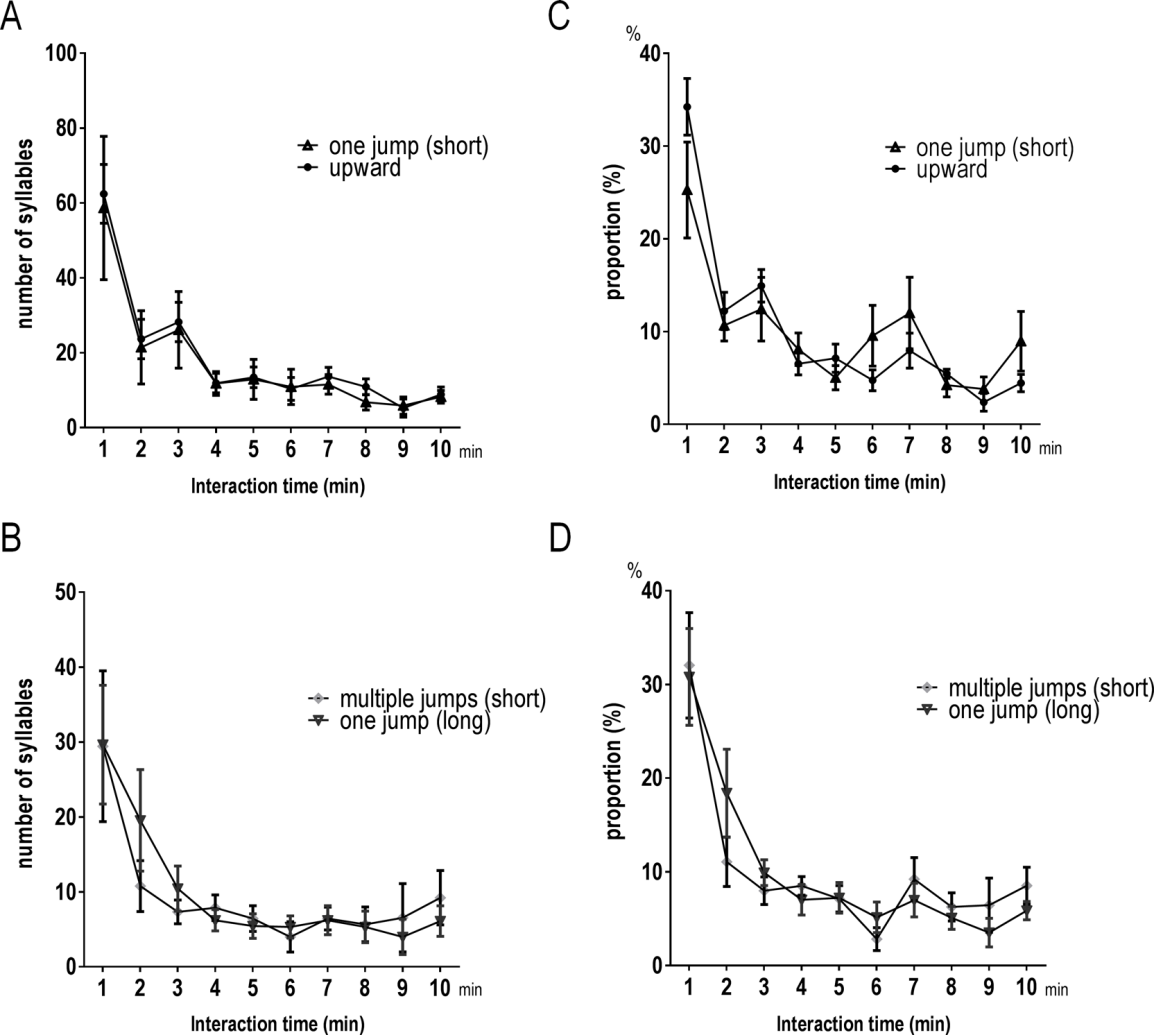
Temporal changes of four syllable types during male–female interaction. Temporal changes in the number of syllables (A and B) and the proportion (C and D) of four syllable types over 10 min. The changes in the number and proportion were similar between Upward and One jump (short), and between One jump (long) and Multiple jumps (short).

We analyzed temporal (per minute) changes in vocalizations during sniffing in nine mice, that displayed this behavior throughout courtship. Several categories of syllables showed similar temporal changes. Temporal fluctuation in the number of Upward syllables produced was similar to that of One jump (short) (*R* = 0.99, *p* < 0.05; Fig 5A), and One jump (long) showed similar changes as Multiple jumps (short) (*R* = 0.91, *p* < 0.05; Fig 5B). These syllable pairs were highly correlated in six of the nine animals (*R* > 0.7, *p* < 0.05). To examine whether all mice showed similar trends in the production of syllables throughout the interactions, we compared the proportions of each syllable type with contact time. We defined the proportion as the number of syllables for 1 min divided by the total number of the syllables. Consistent with the results for number of syllables, proportions were similar and there were no significant differences between Upward and One jump (short) or between One jump (long) and Multiple jumps (short) (Fig 5C and 5D).

**Fig 6:**
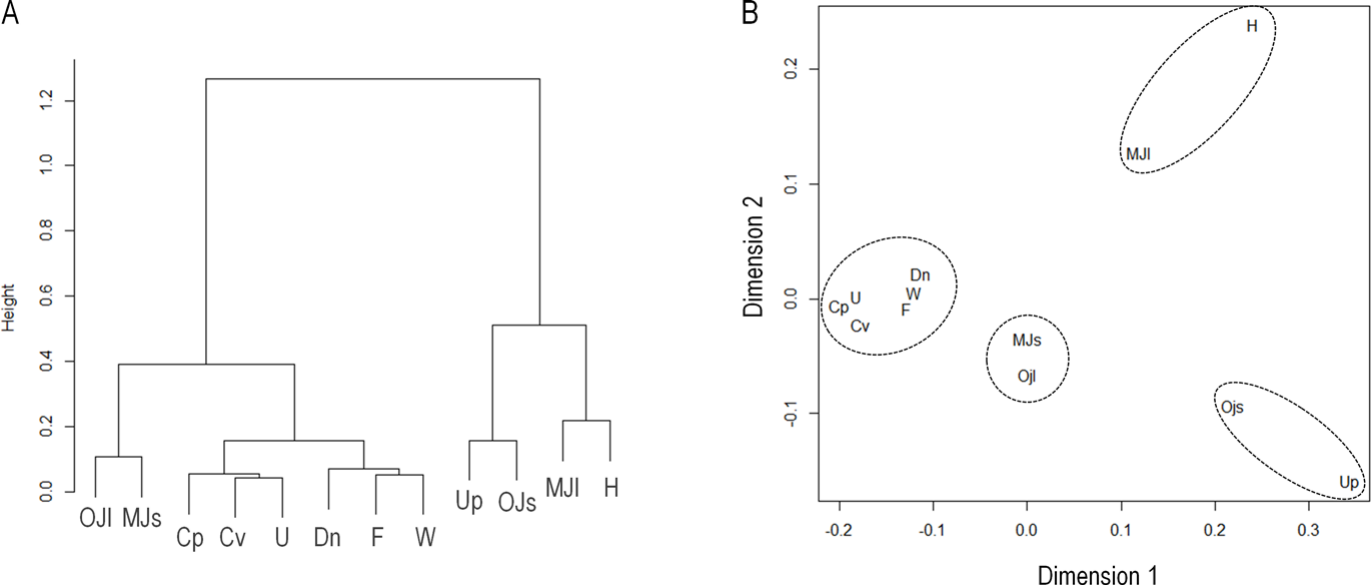
Multidimensional scaling analysis of syllable types. Hierarchical clustering (A) and an MDS plot (B) of context-specific changes in the proportion of each syllable type; Upward (Up), Downward (Dn), Flat (F), Chevron (Cv), U shape (U), Wave (W), Complex (Cp), One jump short (OJs), One jump long (OJl), Multiple jumps short (MJs), Multiple jumps long (MJl), and Harmonics (H).

Changes in vocalizations as the interactions progressed can aid syllable classification. Clustering of proportions of vocalizations in ES, MS, MM, and LS indicated four separate clusters: (1) Upward and One jump short, (2) One jump long and Multiple jumps short, (3) Multiple jumps long and Harmonics, and (4) others (Fig 6A). The MDS plot, which shows the similarity of use between syllable types, also indicates a specific clustering pattern (Fig 6B). There was a high correlation between dimension 1 and the average proportion of syllable types over the course of the interactions (*R* = 0.99, *p* < 0.0001), and between dimension 2 and the inclination of the change in the proportion of the vocalizations featuring simple, complex, and Harmonics syllables (*R* = 0.99, *p* < 0.0001). In addition, dimension 2 was highly correlated with mean syllable duration (*R* = 0.71, *p* < 0.001), peak frequency (*R* = -0.76, *p* < 0.001), and fundamental frequency (*R* = -0.8, *p* < 0.001).

### Difference in ultrasonic vocalizations in the early phase among mice displaying only sniffing or both sniffing and mounting behavior

**Fig 7:**
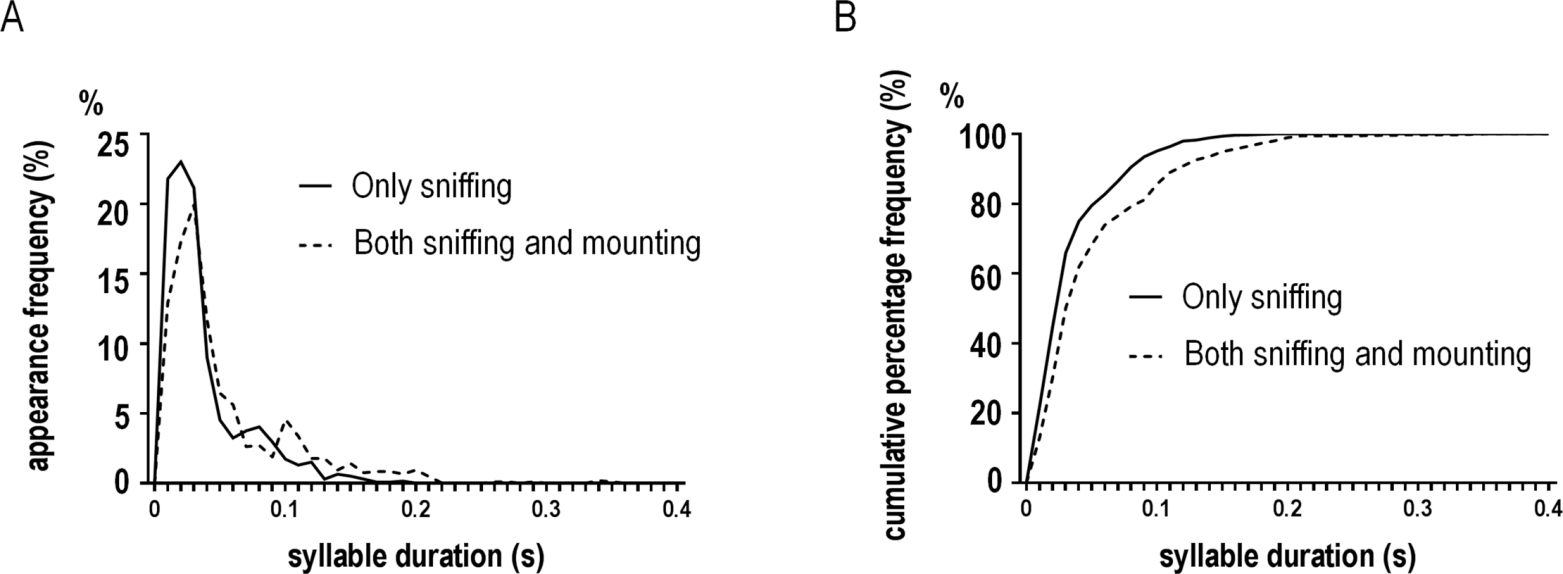
The difference in syllable duration associated with later behavior. Distribution (A) and cumulative percentage (B) of syllable duration during vocalizations in the early phase, between mice that exhibited only sniffing (continuous line) and mice that exhibited both sniffing and mounting behavior (dashed line) over the course of the interaction. Both groups of animals displayed only sniffing behavior in the early phase. The differences between these groups were significant (*p* < 0.01).

We compared vocalizations in the first 30 s of the interaction between animals that showed only sniffing throughout the interaction (1390 syllables, n = 10) and those that showed both sniffing and mounting (1069 syllables, n = 6) to determine whether there were differences in early phase vocalizations between these two groups. Although there were no significant differences in the number of syllables, mean syllable duration, or the proportion of each syllable type, animals that performed only sniffing exhibited different distributions and cumulative percentages of syllable duration than those that performed both behaviors (two-sided Kolmogorov-Smirnov test, *p* < 0.01; Fig 7).

## Discussion

In this study we performed a detailed investigation of the vocal behavior of adult male mice during courtship. We found that the proportions of syllable categories emitted changed depending on contact time and courtship behavior. In summary: (1) males produced Upward and One jump (short) syllables (both short) soon after the introduction of females; (2) the number of long syllables with frequency jumps increased approximately 1 min after the introduction of females; and (3) males produced longer and more complex syllables with harmonics during the phase that featured mounting behavior.

### Male vocalizations during sniffing change depending on contact time

We found that mean syllable duration was short for approximately 1 min following the introduction of females, after which differences between middle and late phases in mean syllable duration were minor. The syllable patterns and number of vocalizations during sniffing in the middle and late phases were similar, suggesting that after the first minute, the pattern of ultrasonic vocalizations emitted during sniffing remained unaffected by contact time. When mice were subjected to amygdala lesions, the proportion of short syllables they produced increased, and both the number of long syllables and extent of mounting behavior decreased [20]. Therefore, it seems likely that vocalizations in the early phase could be connected with early exploratory behavior when unfamiliar individuals are encountered and have no direct involvement in sexual behavior, which is regulated by the amygdala. On the other hand, it is possible that mice with a lesioned amygdala produce fewer vocalizations overall during the middle and late phases because these vocalizations are strongly associated with sexual behavior. In other words, vocalizations during sniffing in the middle and late phases may be specific to sexual behavior even if there is no overtly reproductive behavior, such as mounting.

When males displayed sniffing behavior, they produced short syllables for approximately 1 min after the introduction of females, and tended to produce a wider range of syllables thereafter. However, the duration of the early phase, during which males emitted many short syllables, varied by individual. Mice that demonstrated mounting behavior throughout the interaction showed a wider distribution of syllable duration during the early phase than mice that only displayed sniffing. This suggests that mice that displayed mounting behavior performed an early shift to sexual behavior-specific vocalizations, including long syllables, and that this factor underlies the temporal variability observed during the early phase of interaction.

We also investigated changes in mouse vocalizations with contact time and sexual behavior in males that had at least one previous interactive experience with a female. Previous studies have reported that mouse vocalizations change according to social experience; in particular, isolated males showed different vocalization patterns from males that were housed in groups [13], and males with previous experience with females emitted a greater number of ultrasonic vocalizations than those with no experience [3]. Factors such as differences in social experience and motivational state should therefore be taken into account in future studies. Although our study did not include variation in the males’ social experience, our findings indicated that mouse vocalizations do change depending on interaction time and sexual behavior.

### Ultrasonic vocalizations before, during and after mounting behavior

We found that mice emitted long and complex syllables during interaction phases that featured mounting behavior (mainly the middle phase). Two individuals showed mounting behavior in the late phase, and the patterns of their vocalizations were similar to those in the MM phase, although the syllable count was lower. This suggests that, similarly to vocalizations during sniffing behavior, vocalization patterns when mounting behavior was displayed did not change with contact time after the early phase.

Our findings regarding long syllables, especially those containing harmonics, are consistent with previous studies. It has been shown that males shift to syllables containing frequency jumps and harmonics during mounting behavior [18] and the number of syllables and percentage of harmonic syllables used increase before mounting [19]. In addition, based on our earlier study, we have suggested that longer syllables, such as those in the Multiple jumps and Harmonics categories, are associated with mounting behavior, which is regulated by the amygdala [20]. In the present study, males emitted more Harmonics syllables during the MM phase, and there was a high degree of correlation between mounting and long syllables. Furthermore, female mice can discriminate between complex and simple vocalizations, and they showed a preference for complex vocalizations including Harmonics or frequency jumps [24]. Chabout et al. [24] suggest that complex syllables, such as those containing Harmonics, play an important role in sexual behavior. Similarly, our data imply that these complex syllables are similar to the syllables expressed in MM. Our findings could therefore be evidence of the strong relationship between complex harmonic sounds and courtship behavior.

Mouse strains exhibit differences in the proportion of categorized syllable patterns they emit. Experiments involving cross-fostering between strains have shown that vocalization patterns were innate [14], and the ultrasonic vocalizations of chronically deaf mice were comparable to those of mice with normal hearing [23]. Mouse courtship vocalizations therefore have distinct patterns that are specific to strains, and animals do not require auditory experience to produce them. However, we found that males shifted to longer syllables containing more jumps and harmonics, and this result is consistent with that of a previous study using a different strain [19]. Our results therefore suggest that complex details such as frequency modulations, duration and harmonics in vocalizations may be important for mouse sexual behavior, independent of strain differences.

Males also produced more syllables before mounting the female, and syllable number and duration tended to decrease after shifting to the intromission phase, during which females showed little resistance behavior. This supports the idea that female response is related to male vocalizations. In future studies, we will examine female reactions to such vocalizations.

### Context-specific classification

The “jump” structure has been emphasized in previous studies investigating categorized syllable types, and it is known that C57BL/6 mice emit a high proportion of syllables containing frequency jumps [9, 11, 14]. However, we found that the number of One jump (short) syllables showed similar temporal variability as Upward syllables (short syllables containing an increase in frequency). Notably, there were many continuous Upward and One jump (short) syllables with an upward frequency modulation in the early phase (Fig 4). This suggests that these syllable types play similar roles in courtship. Likewise, One jump (long) and Multiple jumps (short) syllables in the middle phase also showed similar temporal changes, suggesting that these syllables have similar functions. We suggest, therefore, that not only the number of frequency jumps but also syllable duration and frequency modulation should be taken into account when categorizing syllables during the courtship sequence.

Changes in the proportions of Multiple jumps (long) and Harmonics syllables across courtship phases were similar. These syllables are structurally very similar, because Harmonics syllables include Complex, One jump or Multiple jumps elements. In particular, animals emitted many Multiple jumps (long) syllables containing harmonics during phases that featured mounting behavior. Although it is known that mice can discriminate between dissimilar syllable types, the extent to which they can discriminate between similar ones is not well studied [25, 26]. We will examine the differences of roles in courtship between multiple jumps (long) and Harmonics syllables in future studies.

## Conclusion

This study investigated the changes in male mouse vocalizations during courtship. We found that mice produced different ultrasonic vocalizations depending on contact time and behavior, suggesting that vocalizations play different roles in each phase of the courtship sequence. Combined with previous findings regarding the effects of experience on behavior, this implies that we may be able to predict later behavior from early vocalization. Our results also demonstrate the need to record vocalizations for a sufficient amount of time, to assess syllable duration and type, and to further examine the relationship between vocalizations and behavior, to facilitate useful conclusions about mouse vocalizations. We speculate that in the traditional classification method there are functional overlaps between several syllable types. Our findings contribute to the understanding of vocal communication in mice and to future studies regarding mouse vocalizations.

## Supporting Information

S1 Fig Vocalizations during intromission. Total number of syllables per minute (A), mean syllable duration (B) and mean break duration (C) during mounting and intromission in the three animals that exhibited intromission.
(TIF)

S1 Movie Simple syllables. A representative example of mouse courtship vocalizations in the early phase. The female explored the test box and the male approached the female while producing simple syllables.
(MP4)

S2 Movie Complex syllables. A representative example of mouse courtship vocalizations in the middle phase. The female was sniffing in the corner, and the male approached the female while producing complex syllables.
(MP4)

S3 Movie Harmonics syllables. A representative example of mouse courtship vocalizations in the mounting phase. The male produced Harmonics syllables before and during mounting behavior.
(MP4)
