# Supplementary figures and images for: Phase-Specific Vocalizations of Male Mice at the Initial Encounter during the Courtship Sequence
Source: PLoS One. 2016 Feb 3;11(2):e0147102. doi: 10.1371/journal.pone.0147102 (PMC4739514; doi:10.1371/journal.pone.0147102)

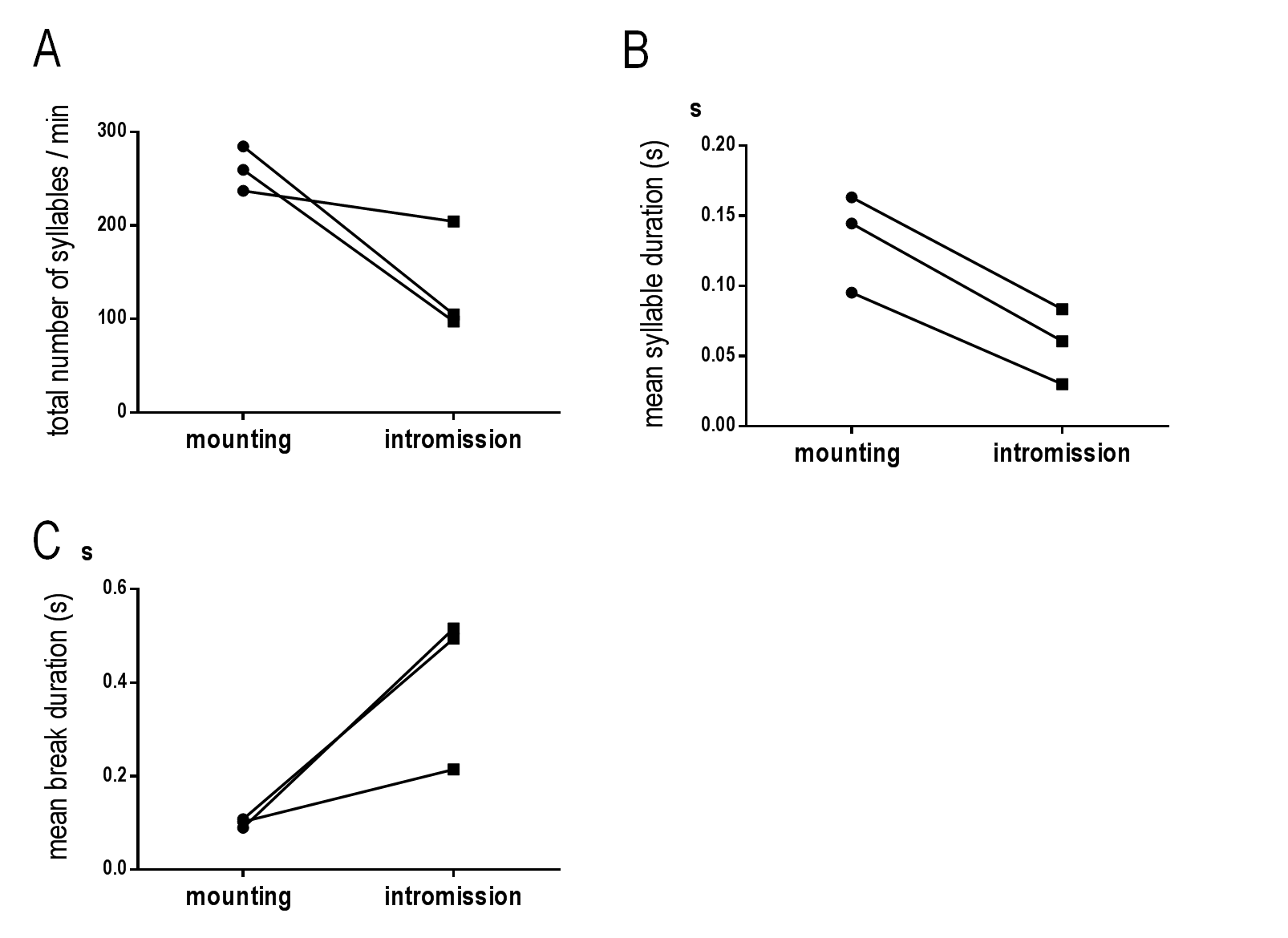

Supplement: S1 Fig — Total number of syllables per minute (A), mean syllable duration (B) and mean break duration (C) during mounting and intromission in the three animals that exhibited intromission. (TIF) [file pone.0147102.s001.tif]
